# AttPNet: Attention-Based Deep Neural Network for 3D Point Set Analysis

**DOI:** 10.3390/s20195455

**Published:** 2020-09-23

**Authors:** Yufeng Yang, Yixiao Ma, Jing Zhang, Xin Gao, Min Xu

**Affiliations:** 1 Computational Biology Department, School of Computer Science, Carnegie Mellon University, Pittsburgh, PA 15213, USA; yfyang16@gmail.com (Y.Y.); mayixiao666@gmail.com (Y.M.); 2 Department of Computer Science, University of California, Irvine, CA 92697, USA; zhang.jing@uci.edu; 3 Computational Bioscience Research Center (CBRC), Computer, Electrical and Mathematical Sciences and Engineering (CEMSE) Division, King Abdullah University of Science and Technology (KAUST), Thuwal 23955-6900, Saudi Arabia; xin.gao@kaust.edu.sa

**Keywords:** point cloud, attention mechanism, deep neural network

## Abstract

Point set is a major type of 3D structure representation format characterized by its data availability and compactness. Most former deep learning-based point set models pay equal attention to different point set regions and channels, thus having limited ability in focusing on small regions and specific channels that are important for characterizing the object of interest. In this paper, we introduce a novel model named Attention-based Point Network (AttPNet). It uses attention mechanism for both global feature masking and channel weighting to focus on characteristic regions and channels. There are two branches in our model. The first branch calculates an attention mask for every point. The second branch uses convolution layers to abstract global features from point sets, where channel attention block is adapted to focus on important channels. Evaluations on the ModelNet40 benchmark dataset show that our model outperforms the existing best model in classification tasks by 0.7% without voting. In addition, experiments on augmented data demonstrate that our model is robust to rotational perturbations and missing points. We also design a Electron Cryo-Tomography (ECT) point cloud dataset and further demonstrate our model’s ability in dealing with fine-grained structures on the ECT dataset.

## 1. Introduction

Point cloud is a main type of geometric data representation of 3D structures. In addition to techniques such as photogrammetry, the rapid development of sensors such as Velodyne spinning Light Detection and Ranging (LIDAR) and tilting laser scanner also makes it drastically easy to collect structural information of the real world using point clouds. This results in broad applications of the combination between photogrammetry and laser scanning techniques. For example, the authors of [1] integrate photogrammetry and laser scanning techniques to model digital 3D dinosaur footprints. Point clouds are easy to learn from because of their expressive and compact representation [2]. Furthermore, compared with volumetric image representations, point cloud takes up significantly less storage when representing the same structure.

In recent years, deep neural network has become a major tool for image analysis. Deep learning is also increasingly popular for analyzing point set data due to its large scale learning capacity. Since the invention of PointNet [3], which directly handles point sets, most recent works extract the global features of a point set by grouping and aggregating features of all the individual points. However, they are limited to detecting the structural differences between distinct objects. Therefore, when confronting similar and complicated structures, the above models may not perform well on classification and segmentation tasks.

**Figure 1:**
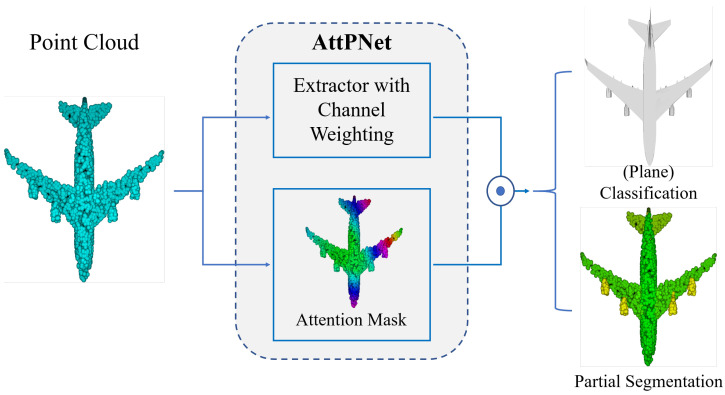
Overview of AttPNet.

**Figure 2:**
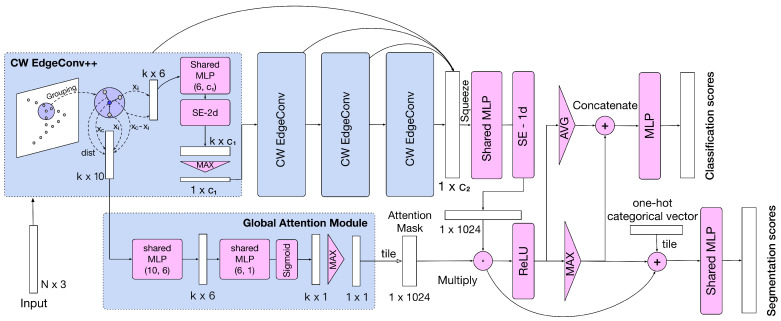
The architecture for classification. This model takes *N* points as an input and mainly contains two branches. The upper branch functions as a regular network to output the global feature. The lower branch is a global attention module which outputs the global attention mask representing different importance of each point. We directly operate an element-wise multiplication between the feature and mask. Finally, we feed the outcome of this multiplication into the classification and segmentation network to obtain the scores. $c_{1}$ and $c_{2}$ denote the dimensions of features. *k* represents the quantity of points $x_{i}$ sampled in a ball centered at $x_{c}$. *m* denotes the number of classes. The “++” of “CW EdgeConv++” means that there is additional output from it for the global attention module. The dimensions annotated in CW-EdgeConv and Global Attention Module (all blue boxes) are for per-point features.

For this reason, we aim at specifically handling fine-grained structures by proposing a novel deep learning model named AttPNet (Figure 1). The model is characterized by attention-based global feature masking and channel weighting which correspond to the global attention module and CW-EdgeConv (see Figure 2). The whole end-to-end model (Figure 2) takes *N* points as an input and learns a global feature for classification and segmentation tasks. There are two main branches in the model. The first branch focuses on the effect of each local point, therefore outputting a global mask at the end of the branch, weighting the contribution of each point to the analysis task. In order to focus on the most discriminative regions of the input structure, we multiply the global feature by the mask to obtain our final attention-based feature. Another branch outputs global geometric information in the form of a two-dimensional tensor by concatenating each point’s feature. We use channel weighting in this branch to focus on informative and distinct channels.

Experiments show that our model outperforms existing models on the most widely used ModelNet40 benchmark dataset. Note that on the ModelNet40 leaderboard, the 93.6% result of RSCNN trains multiple models to vote for the final decision. For a fair comparison, following the practice of most of deep learning papers, we compared our method with other models on ModelNet40 without voting. The key reason why our work outperforms other models is that we innovatively introduce the attention mechanism to point cloud feature extraction. Former models like PointNet [3] and PointNet++ [4] do not distinguish the importance of each point. However, every point plays a unique role in characterizing the overall structure. Therefore, we let our model assign every single point its own weight in the feature integration phase. Moreover, the squeeze-and-excitation operation [5] used for the channel attention in every convolutional layer also makes the model focus on the important channels of features that representing the internal geometric information in high dimensional space.

Our main contributions are summarized as follows.

- We propose a novel model named AttPNet which uses attention mechanism for both global feature masking and channel weighting to focus on characteristic regions and channels.
- Our model achieved 93.6% accuracy of overall instances on ModelNet40 benchmark dataset without voting and outperforms the existing best point set model by 0.7%. Given that the performance improvement is slow in recent years, the performance improvement of our model is significant.
- Experiments show that our model generalizes better on test data with random translation, rotation, and missing points perturbations Table 1.

## 2. Related Work

### 2.1. Point Cloud Networks

#### 2.1.1. Projection and Voxelization

Before the invention of PointNet [3], the deep learning methods for point clouds can be divided into several types. The most important two techniques are projection and voxelization. The authors of [6,7,8] project 3D point clouds into 2D images from multiple angles of view and feed 2D images into traditional 2D convolutional layers. These approaches dominate for a long period due to efficiency but they are limited by the problem of occluded objects. The authors of [9] propose a method that partially solve object occlusion by aggregating different views from sensors. Voxelization [6,10,11,12] is also a popular type of approach that subsample point clouds into volumetric grids so as to utilize 3D convolutional layers. Such methods are mainly constrained by the inflexible resolution and high computational and storage cost. The authors of [13] propose a novel solution similar to voxelization which projects the point clouds into high-dimension lattice and applies bilateral convolution layers [14]. Splatnet achieves competitive outcomes on several data sets compared with pointnet++ [4]. Octnet [15] use unbalanced octrees to hierarchically partition the space through exploring the sparsity in 3D volumetric data. Each leaf node of the unbalanced octree stores a pooled feature representation.

#### 2.1.2. PointNet & PointNet++

PointNet [3] is a pioneering work that directly consumes point clouds and utilizes symmetric functions such as max pooling to respect the permutation invariance of points. It is highly efficient and achieved better results than previous work. PointNet++ [4] is an improved version of PointNet. By applying hierarchical abstraction layers, it is capable of learning local features with increasing contextual scales and has significantly better results on several benchmark datasets than PointNet.

#### 2.1.3. Graph Networks

The authors of [16] propose a new module (EdgeConv) which acts on graphs dynamically computed in each layer of the network. The design of the dynamic graph module can also learn both local neighborhood information and global shape properties. The architecture of AttPNet model is mainly based on dynamic graph network (DGCNN). Key differences between AttPNet and DGCNN include the extension of original EdgeConv and global attention module. Apart from these two distinct differences, we made some minor structural adjustments to the network such as feature dimension, number of module and the selection of activation function. The authors of [17] use recursive feature aggregation on a nearest-neighbor graph computed from 3D positions to generate local high-dimensional features and also defines a point-set kernel in analogy to 2D convolution kernels for images. The authors of [18] propose a model named GS-Net to deal with data rotation and translation. It adopts Eigen-Graph to collect geometric information from points in a distance. For points in neighbors, this algorithm combines both Euclidean space and Eigenvalue space to generate features.

#### 2.1.4. Point Convolution

Recently, there is an increasing interest in designing convolutions that directly operates on point clouds, inspired by the great performance of CNN on 2D images. To design a point convolution network, the authors of [19,20,21] attempt to construct continuous kernel functions to convolve on local points. PointConv [19] uses a Multi-Layer Perceptron (MLP) to fit a kernel due to its ability to approximate an arbitrary continuous function. It also consumes the inverse density as a feature to convolve with the kernel function. SpiderCNN [20] found that the MLP did not work well on approximating the kernels, so the authors propose the order-3 Taylor term which is a family of polynomial functions applied with different weights to enrich the complexity of the filters. Flex convolution [21] utilizes linear functions to act as a kernel which is actually an order-1 Taylor term of SpiderCNN. Structure-aware Convolution (SAC) [22] matches neighbor points in the point cloud through 3D convolution to extract geometric features. These convolution works all have significant improvements in several data sets but the training and inference time are much longer than PointNet++ (usually double).

Although networks like SpiderCNN [20] and DGCNN [16] incorporate local neighborhood information, these extraction steps are region-wise. Strategies mentioned above work well on classification tasks between distinctive categories. However, they only consider the global and neighbor effect between groups of points but ignore the location and other hidden information of a single point. Our approach, AttPNet, has a point-wise branch to solve this problem.

#### 2.1.5. Sequence Network

The authors of [23] employ a sequence model to capture the correlations by aggregating multi-scale areas of each local region with attention. Point2Sequence utilized LSTM [24] as the main module of the encoder and decoder to highlight the importance of different area scales. However, due to the introduction of LSTM, the model is hard to train and needs more time to converge.

### 2.2. Attention-Based Methods in Computer Vision

The attention mechanism has been well studied in computer vision and help achieve great improvement in scene analysis [25]. From the era of deep learning, attention mechanism was widely known because of a sequence model [26] in translation to focus on key words in natural language processing. In recent years, it also demonstrated useful in extracting the core information in images. Such approaches can be divided into hard attention and soft attention. The work in [27] uses a classical method of hard attention. The author proposes an APM module to focus and crop the distinct area in fine-grained classification tasks. This hard-attention module only acts in looking again (in comparison to look once in YOLO model) at the crucial area and ignoring all other pixels. To resolve the problem of non-differentiable cropping, many researchers attempt to utilize the soft attention mechanism which learns an alignment weight and place it on all pixels such as [28,29]. From another perspective, the attention mechanism can also be separated into two parts: spatial-domain attention and channel-domain attention. SENet [5], as a championship winner in 2017 ILSVR, utilized SE block, which can be regarded as a channel-domain attention module that adaptively recalibrates channel-wise feature responses by explicitly modeling interdependencies between channels. SENet produces significant performance improvements at little computational cost and initiates the methods on channel-wise recalibration and attention.

There are also few works using the idea of attention mechanism to improve the results on classification and segmentation. The work in [30] includes a simple contextual modeling mechanism to automatically select contextual region and aggregate features. The work in [31] uses a parameter-efficient Group Shuffle Attention (GSA) and develops Point Attention Transformers (PATs) to construct an end-to-end learnable model. The work in [32] introduces a geometry-attentional network which combines features from geometry-aware convolution, attention module and other hierarchical architectures. The work in [33] proposes an local relation learning module based on the attention mechanism in order to extract local features. However, the improvement of these works on point cloud datasets such as ShapeNet and ModelNet40 are limited. The best classification result of the work in [30] on ModelNet40 is 90.0% and best part segmentation result is 84.6% (mean class accuracy) which are both lower than the results of PointNet++ [4]. For the model [31], the classification result on ModelNet40 is 91.7%. The authors of [34] use a Graph Attention Convolution (GAC) to solve semantic segmentation tasks, but their attention mechanism is based on the subgraph of a point cloud and only accept neighbor feature as input. In contrast, our model applies a different design of attention mechanism which combines global feature and channel feature during training process and gains significant improvement.

## 3. Method

In this section, we first describe the CW-EdgeConv and the global attention module. Then, we overview the whole model for classification and segmentation. Finally, we compare several structures of attention modules.

### 3.1. Channel Weighting Edge Convolution (CW-EdgeConv) Module

Our CW-EdgeConv module is an extension of EdgeConv and it consists of four steps: (1) calculate k nearest neighbors using kNN query, (2) map low-dimensional geometrical features to high-dimensional features using Multilayer Perceptron (MLP) [35], (3) channel weighting, and (4) aggregate features of nearest neighbor points into features of a single point. The original EdgeConv will be described in the last of this subsection.

The first step is kNN query, which inputs a set of points and calculates the k nearest neighbors for each point. Specifically, consider an point set input $x=\left\{x_{i}|\;i\in \left[1,N\right]\right\}\in R^{N\times C}\}$, where *N* is the total number of points and *C* is the dimension of geometrical features of a point, such as 3D location and normal. Given that our model does not resample points before each CW-EdgeConv layer, the number of points considered remains *N*. For each $x_{i}$, we define a subset centered at it and choose k−1 nearest points except the center $x_{c}$. Therefore, a kNN query of $x_{c}$ can be calculated as

(1) $$\begin{array}{c} F_{r}\left(x_{c}\right)=\{x_{j}\;|\;∥x_{j}-x_{c}{∥}_{2}\leq ∥x_{c}-x_{k}{∥}_{2}\}\in R^{k\;\times \;C} \end{array}$$

where $x_{k}$ is the *k* th nearest point from $x_{c}$, calculated using the kNN query. Therefore, the grouped input can be represented by

(2) $$\begin{array}{c} \left\{F_{r}\left(x_{i}\right)\;|\;x_{i}\;\in \;x\right\}\;\in \;R^{N\times k\;\times \;C} \end{array}$$

We apply the kNN query method to group the point set in each layer due to simplicity and less inference time.

The second step is using MLP to map low dimensional geometrical features to high-dimensional features. These low dimensional geometrical features include the edge feature in form of $x_{j}-x_{i}$ and the original input points $x_{i}$, where $x_{j}\in F_{r}\left(x_{i}\right)$. The choice of such features strictly follows the best option in EdgeConv [16]. Given such features, we use MLP to calculate high-dimensional features. Specifically, we apply a 2D 1×1 convolutional layer followed by a batch normalization layer [36] and a ReLU activation function [37]. We use the following notation to represent this convolutional operation of one group.

$$\begin{array}{c} h_{\Theta }\left(x_{j}-x_{i},x_{i}\right),\;x_{j}\;\in \;F_{r}\left(x_{i}\right) \end{array}$$

Note that $h_{\Theta }$ is shared through all groups in that it works as a nonlinear function to discover the intrinsic features of each group in high dimensional space such as density, mean distance, etc. This is achieved by extracting the correlation of the input geometric features $\left(x_{j}-x_{i},x_{i}\right)$.

**Figure 3:**
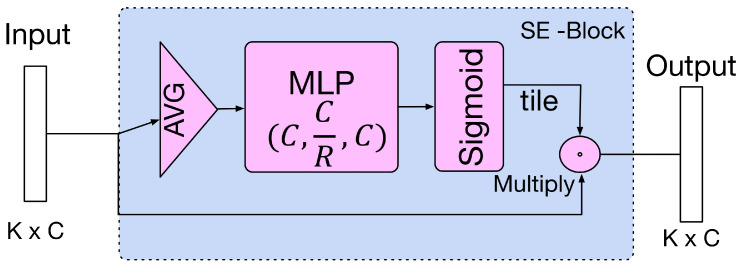
SE-Block architecture.

For the third step, given the middle features outputted from convolutional layers $h_{\Theta }$, we apply channel weighting on these middle features by adapting a squeeze and excitation block (SE-Block2d) [5] layer. The architecture of SE-Block is shown in Figure 3. Here, we simply abbreviate the SE-Block as $F_{\mathrm{se}}$.

(3) $$\begin{array}{c} x^{\mathrm{se}}=F_{\mathrm{se}}\left(h_{\Theta }\left(x_{j}-x_{i},x_{i}\right)\right)\in R^{N\times k\;\times \;C^{\mathrm{out}}},\;x_{j}\;\in \;F_{r}\left(x_{i}\right) \end{array}$$

where $C^{\mathrm{out}}$ is the number of the output channels of $h_{\Theta }$.

We made two modifications to the SE-Block [5]: (1) We adapt a 1d channel weighting model to fit the dimension of the concatenated feature; (2) We keep the original channel size of a layer in the block because the reduction of layer parameters limits the performance of channel weighting.

In the fourth step, we aggregate features of k nearest neighbor points $F_{r}\left(x_{i}\right)$ into features of a single point $x_{i}$. This is similar to 2D convolution networks that each pixel value should be aggregated from several values of a kernel. Here, we follow the convention of PointNet, PointNet++, and EdgeConv; the aggregation function is Max(·) instead of ∑. The output for a group centered at $x_{i}$ is calculated as follows.

(4) $$\begin{array}{c} x_{i}^{\mathrm{CW}}=\max_{j\;\in \;[1,\;k]}x^{\mathrm{se}}\left(x_{i},\;x_{j}\right),\;x_{j}\;\in \;F_{r}\left(x_{i}\right),\;x_{i}\;\in \;x \end{array}$$

Finally, the output of the whole CW-EdgeConv is calculated as follows.

(5) $$\begin{array}{c} x^{\mathrm{CW}}=\left\{x_{i}^{\mathrm{CW}}\;|\;i\;\in \;\left[1,\;N\right]\right\}\;\in \;R^{N\;\times \;C^{\mathrm{out}}} \end{array}$$

We denote the output of the *l* th CW-EdgeConv layer as ${}^{\left(l\right)}x{}^{\mathrm{CW}}$.

After the output ${}^{\left(4\right)}x{}^{\mathrm{CW}}$ of the last CW-EdgeConv layer, we further utilize an shared MLP $h_{\Theta }^{g}$ and a SE-1d block to obtain the global feature g.

(6) $$\begin{array}{c} g=F_{\mathrm{se}}\left(h_{\Theta }^{g}\left({}^{\left(4\right)}x{}^{\mathrm{CW}}\right)\right)\;\in \;R^{N\;\times \;C^{\mathrm{out}}} \end{array}$$

Remarks: The only difference between first CW-EdgeConv++ layer and following CW-EdgeConv layers is that there are additional geometric features for the global attention module (see more detail in the next subsection). The form of this additional output is represented as

(7) $$x_{i}^{{\mathrm{CW}}_{2}}=\{x_{i},\;x_{j},\;x_{j}-x_{i},\;∥x_{j}-x_{i}{∥}_{2}\}\;\in \;R^{k\;\times \;10}$$

where $x_{i}\;\in \;x$, $x_{j}\;\in \;F_{r}\left(x_{i}\right)$, ${∥\cdot ∥}_{2}$ denotes the euclidean distance, and *k* specifies the number of points in a group.

Remarks: The original EdgeConv module only contains step 1, 2, and 4 of CW-EdgeConv. Compared with Equation (4), the output $x_{i}^{\mathrm{EC}}$ for a group centered at $x_{i}$ in EdgeConv can be calculated as

(8) $$\begin{array}{c} x_{i}^{\mathrm{EC}}=\max_{j\;\in \;[1,\;k]}h_{\Theta }\left(x_{j}-x_{i},x_{i}\right),\;x_{j}\;\in \;F_{r}\left(x_{i}\right),\;x_{i}\;\in \;x \end{array}$$

### 3.2. Global Attention Module

The input of this module is the output $x_{i}^{{\mathrm{CW}}_{2}}$ of the CW-EdgeConv++ module (see Figure 2). Similar to the channel attention in SENet [5], we utilize two 1×1 2D convolutional layers to reduce the dimensions of grouped features (the input of this module) and one sigmoid function to generate the soft attention mask (Figure 2). For a specific point group $F_{r}\left(x_{i}\right)$ centered at $x_{i}$, the importance $x_{i}^{\mathrm{GA}}$ is calculated as

(9) $$\begin{array}{c} x_{i}^{\mathrm{GA}}=\max_{j\;\in \;[1,\;k]}\mathrm{Sigmoid}\left(h_{\Theta_{2}}\left(x_{i}^{{\mathrm{CW}}_{2}}\right)\right)\;\in \;R^{1\;\times \;1} \end{array}$$

where the number of output channels of $h_{\Theta_{2}}$ is one and Sigmoid denotes the sigmoid activation function which is $\frac{1}{1+e^{-x}}\in \;\left(0,\;1\right)$. Finally, the module outputs a learned soft mask $x^{\mathrm{GA}}=\left\{x_{i}^{\mathrm{GA}}|\;i\in \left[1,\;N\right]\right\}$.

The motivation of this design is simple: We consider the classification task as an example. Each object class has its characteristic patterns that make it distinct from other classes. Examples of such characteristic patterns include the string of guitars, the wings of airplanes, etc. Such characteristic patterns may be neglected due to excessive amount of features extracted during the pooling aggregation process. Therefore, it is necessary to measure the importance $x_{i}^{\mathrm{GA}}$ of each group $F_{r}\left(x_{i}\right)$ and use such $x_{i}^{\mathrm{GA}}$ to weight the global feature g by our learned soft mask $x^{\mathrm{GA}}$.

The reason why we feed more pivotal geometric information (i.e., $∥x_{j}-x_{i}{∥}_{2}$ in Equation (7)) into the global attention module is to accelerate and improve the learning of the global soft mask $x^{\mathrm{GA}}$. Though MLP can approximate any nonlinear functions theoretically such as high-order information like the square of the euclidean distance (2-order: $∥x_{j}-x_{i}{∥}_{2}^{2}$) from a group, experiments show that the model with high-order convolutional filters such as $\left(\omega_{1}x+\omega_{2}x^{2}+\omega_{3}x^{3}\right)$ can achieve higher classification accuracy in several benchmarks [20]. To resolve this same problem in our model, inspired by this idea, we here feed additional pivotal geometric information (i.e., $∥x_{j}-x_{i}{∥}_{2}$ in Equation (7)) to assist the shared MLP to efficiently find features of characteristic patterns and determine the importance $x_{i}^{\mathrm{GA}}$ of every input point $x_{i}$.

In summary, this module aims at automatically discovering the characteristic patterns of point clouds and generate a point-wise soft attention mask $x^{\mathrm{GA}}$ to multiply the global feature g.

### 3.3. Architecture for Classification and Segmentation

After obtaining the mask $x^{\mathrm{GA}}$ from the global attention module and the global feature g, we operate an element-wise multiplication between them and utilize the ReLU activation function to generate the new global feature $g^{m}$ denoting the g after being masked.

For classification (Figure 2), we use both max-pooling and average-pooling to aggregate all points in global feature $g^{m}$ and concatenate them. Finally, we use a 3-layer MLP to output the classification scores. C, C/R, and C are dimensions of three neural layer of the MLP, respectively, where R is the reduction factor to reduce the amount of parameters.

For segmentation, similar to other approaches, we first tile the one-hot category label and concatenate it with the global feature $g^{m}$ and the output of ReLU and max-pooling on $g^{m}$ (Figure 2). The following 4-layer MLP eventually outputs the point-wise segmentation scores.

The selection of aggregation function through all points was actually discussed in a few researches [16]. Most models use the max-pooling other than average-pooling layer due to the convention inherited from PointNet. Intuitively, the max-pooling ought to be better than avg-pooling because the strongest activation is probably the most prominent feature of one class. However, the outcome of avg-pooling can also reflect an important trait of a class; otherwise, the models using avg-pooling will not have a reasonable result. In order to gather more valuable information, we choose to concatenate both results from avg-pooling and max-pooling layers into a complete vector for classification whose dimension is 2048.

### 3.4. Alternative Attention Modules

**Figure 4:**
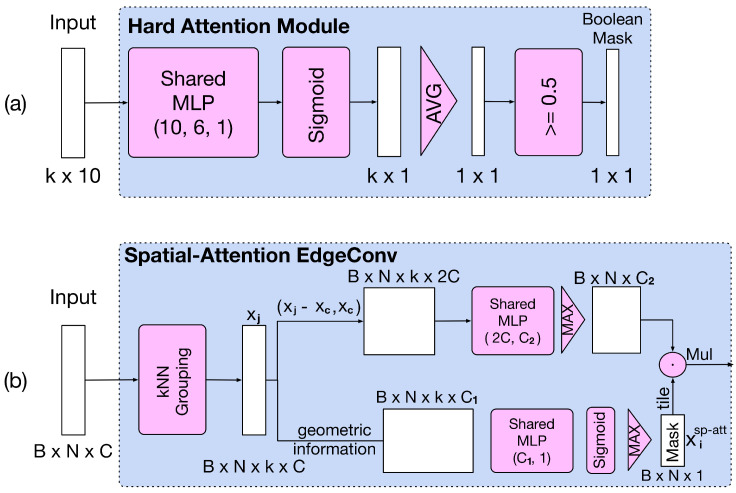
Different structures of attention mechanism. (**a**) Global-attention module with hard-attention. (**b**) EdgeConv with spatial attention.

Inspired by convolution neural network models on 2D images and sequence models, we propose two other modules of attention mechanism (Figure 4) on point clouds as follows.

#### 3.4.1. Global Hard-Attention Module

From Figure 4a, we can see that the global hard-attention module is similar to the one in Figure 2. The input is still represented by $x_{i}^{{\mathrm{CW}}_{2}}$ as mentioned above. First, we use shared MLP and sigmoid function to condense the high-dimensional geometric features. Then, we apply an average-pooling layer to extract the mean response of all features in a group.

(10) $$\begin{array}{c} {\bar{x}}_{i}^{{\mathrm{CW}}_{2}}=\underset{j\in [1,\;k]}{\mathrm{avg}}\mathrm{Sigmoid}\left(h_{\Theta }\left(x_{i}^{{\mathrm{CW}}_{2}}\right)\right) \end{array}$$

In order to suppress all redundant points, we construct the boolean mask $x^{\mathrm{HA}}$ by comparing the ${\bar{x}}_{i}^{{\mathrm{CW}}_{2}}$ and 0.5 as below.

(11) $$\begin{array}{c} x_{i}^{\mathrm{HA}}={\bar{x}}_{i}^{{\mathrm{CW}}_{2}}\geq 0.5\;\;\in \;\left\{0,1\right\} \end{array}$$

Before operating the element-wise multiplication, we expand the channel dimension of the boolean mask $x^{\mathrm{HA}}=\left\{x_{i}^{\mathrm{HA}}\;|\;i\in \left[1,\;N\right]\right\}$ as the same size of the global feature g.

#### 3.4.2. Spatial-Attention Edgeconv

As the global attention module only takes into account the low-dimensional geometric information, the performance may be limited by the lack of high-dimensional intrinsic features in all groups. Therefore, we propose a Spatial-Attention EdgeConv module by further integrating point-wise attention with EdgeConv, as shown in Figure 4b.

Specifically, following the notation in the CW-EgdeConv subsection, we have the input point set x. Furthermore, we denote the $F_{r}\left(x_{c}\right)$ as the group centered at $x_{c}$ by kNN query method. The output of the upper branch of Figure 4b is written as following, which is identical to EdgeConv.

(12) $$\begin{array}{cc} \; & x_{i}^{\mathrm{EC}}=\max_{j\;\in \;[1,\;k]}h_{\Theta }\left(x_{j}-x_{i},\;x_{i}\right),\;x_{j}\;\in \;F_{r}\left(x_{i}\right),\;x_{i}\;\in \;x \end{array}$$

(13) $$\begin{array}{cc} \; & x^{\mathrm{EC}}=\left\{x_{i}^{\mathrm{EC}}\;|\;i\;\in \;\left[1,\;N\right]\right\}\;\in \;R^{N\times C^{\mathrm{out}}} \end{array}$$

In the lower branch of Figure 4b, we first concatenate geometric information represented by $c(x_{i})$. Unlike the first CW-EdgeConv++ layer in Figure 2, $c(x_{i})$ does not include the euclidean distance due to the problem of gradient explosion. In practice, we found that the loss would become NaN after several mini-batches because of the numerical instability when computing the gradient of high-dimensional distance. We calculate the point-wise soft mask $x_{i}^{\mathrm{sp}\text{-}\mathrm{att}}$ as follows.

(14) $$\begin{array}{cc} \; & x_{i}^{\mathrm{sp}\text{-}\mathrm{att}}=\max_{j\in [1,\;k]}\mathrm{Sigmoid}\left(h_{\Theta }\left(c\left(x_{i}\right)\right)\right) \end{array}$$

(15) $$\begin{array}{cc} \; & x^{\mathrm{sp}\text{-}\mathrm{att}}=\left\{x_{i}^{\mathrm{sp}\text{-}\mathrm{att}}\;|\;i\in \left[1,\;N\right]\right\} \end{array}$$

In our implementation, $h_{\Theta }$ contains a batch norm layer after the shared MLP.

Finally, combining the output of the two branches, the output of this whole module is calculated as

(16) $$\begin{array}{c} x^{\mathrm{SA}}={\tilde{x}}^{\mathrm{sp}\text{-}\mathrm{att}}\cdot x^{\mathrm{EC}} \end{array}$$

where ${\tilde{x}}^{\mathrm{sp}\text{-}\mathrm{att}}$ represents the soft mask $x^{\mathrm{sp}\text{-}\mathrm{att}}$ being expanded as the same size of $x^{\mathrm{EC}}$. Though the amount of parameters of Spatial-Attention EdgeConv seems to be less than CW-EdgeConv, the computational cost is more expensive than the $F_{\mathrm{se}}$ operation and the performance is also inferior, as shown from the experiments (see Table 2).

## 4. Experiment

### 4.1. Implementation Details

Our models are implemented in Pytorch. All training and testing experiments run on a single GPU (GTX 1080 Ti). We utilize the SGD optimizer with 0.03 initial learning rate and cosine annealing scheduler ($T_{max}=$ training epochs & minimum learning rate = 0) [42]. Our models often reach 91.0% within three hours and approximately take 16∼18 h on ModelNet40 to converge and achieve best results. The channel size of four EdgeConv layers (1 CW-EdgeConv++ and 3 CW-EdgeConv) for classification are (64,64,128,256) sequentially.

### 4.2. Classification Results

#### Datasets

In the task of classification, we evaluate on several datasets ModelNet40 [10] and Electron Cryo-Tomography (ECT) [43]. ModelNet40 is a dataset made up of 40 common object categories with 100 CAD models per category, among which all the point sets are augmented by scaling, translation, and shuffling. The single-particle ECT [43] dataset consists of 3D images of seven classes of macrocellular structures. We apply constant sampling to generate 400 point cloud data for each class. Compared with other general point cloud dataset, the structures between different classes in ECT dataset are more similar to each other.

### 4.3. ModelNet40

In Table 2, we compare our model with existing state-of-the-art models on ModelNet40 datasets. For a fair comparison, we strictly follow the technique of training and data augmentation in DGCNN (translation, scale and shuffle). Besides, we forsake the voting test because decision by multiple models will largely increase the cost of time and space and conceal the real capability of a single model. Results showed that our (global+ channel attention) model achieves state-of-the-art (93.6%) when the input is 1024 points without majority voting. Other models with different attention mechanism also achieved improvement compared with existing state-of-the-art models with 1024 input points. (BASELINE) represents the baseline model which contains only EdgeConv (no CW-EdgeConv and global attention module). The architecture of this baseline model is slightly different from the DGCNN [16]. (*multi-attention*) means that every Spatial-Attention EdgeConv layer is substituted for CW-EdgeConv and Global-Attention is removed. (*hard-attention*) represents the model only using the Hard-Attention module.

As shown in Table 2, the overall accuracy (OA) of our baseline model reaches 92.5%. Only with the extension of global attention module which increasing very few parameters, the model can achieve 92.9% OA. Replacing all EdgeConv with CW-EdgeConv and retaining the global attention module, the model performs 1.1% better than our baseline.

Actually, Spatial-Attention EdgeConv shares the same idea with Global-Attention module except for the number and location of masking. In order to carry out the ablation study of such attention mechanism, we first remove all SE-Blocks and the Global-Attention Module in Figure 2. Then, we replace different number of common EdgeConv with Spatial-Attention EdgeConv (from left to right in Figure 2) and compare the results in Table 3. From the outcomes, when the number of replacements is 0, which means only common EdgeConv in our model, the result reach 92.8%. Moreover, we find the model numbers 1 and 4 generate better results than the others. This inspire us that probably the fundamental geometric information extracted right after the first layer and the masking on the global feature are more important, thus prompting us to design the Global-Attention Module which can achieve a best trade-off between accuracy and complexity of time and space.

### 4.4. ECT

In the test on ECT dataset (Table 4), our model achieved 96.28% accuracy with global attention and channel weighting. By contrast, PointNet poorly classified fine-grained structures with only 47.78% accuracy probably because it only has global feature aggregation and does not extract local features. PointNet++ achieved 94.62% when integrated the hierarchical local feature extraction. Thanks to the attention mechanism to focus on distinct parts of macro-molecules, our model outperforms existing methods and achieves 96.28%.

### 4.5. Part Segmentation Results

#### 4.5.1. Dataset

We evaluate our models on ShapeNet for part segmentation. The ShapeNet dataset contains 16 categories of objects and consists of 50 different parts in total. Each category has been annotated with two to six parts unequally. The training and testing 3D point sets are 14,006 and 2874, respectively. The aim is to assign every point a part label from 0 to 49. The two evaluation metrics we used are the mean IoU of 16 classes and all instances same as in [3,4,13,19].

#### 4.5.2. Shapenet

The sizes of 4-layer MLP channels in our segmentation model are 256, 256, 128, and 50. The number of the last channel is the amount of part labels in ShapeNet. In the training phase, we used 16 batch size and consumed approximately 10 G memory on GPU. The total training time is slightly more than that in classification. From Table 5, we can see that our model achieves competitive results comparing to the models with additional input (normal vectors). The mean IoU per class is 82.8% and per instance is 85.2%.

Considering the slight difference of architecture between AttPNet and DGCNN, it is evident that our CW-EdgeConv and global attention module have impact on the performance of whole model. Compared with minor improvement between previous state-of-the-art models (see Table 5), AttPNet achieves 0.5% improvement in class mIou than DGCNN.

### 4.6. Visualization of Attention

**Figure 5:**
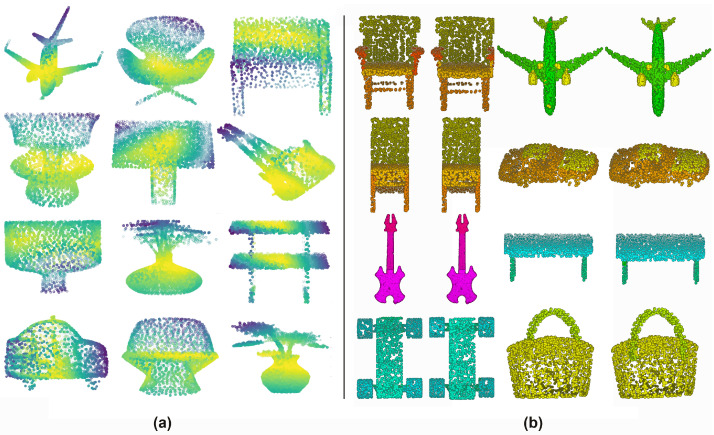
(**a**) Visualization of the attention mask on several point clouds of ModelNet40 dataset. The color changed from dark to light represents the weight from 1 to 0. (**b**) Visualization of part segmentation results on ShapeNet. We visualize some part segmentation results on ShapeNet across several categories. The left of each pair is the prediction of our model and the right is the groundtruth.

We visualize the global-attention mask on point clouds of ModelNet40 dataset. In Figure 5, the color from dark to light represents the soft-attention weight from high to low. Therefore, the darker area is the focus of our global-attention model. We can see that our model underlines the corner and boundary of objects such as airplanes, desks, chairs probably because the point groups of these regions have unique geometric information. Furthermore, our model automatically focuses on such characteristic regions as the strap of bags and the flowers of a vase which make them distinct from other classes.

### 4.7. Robustness

#### 4.7.1. Missing Points

**Figure 6:**
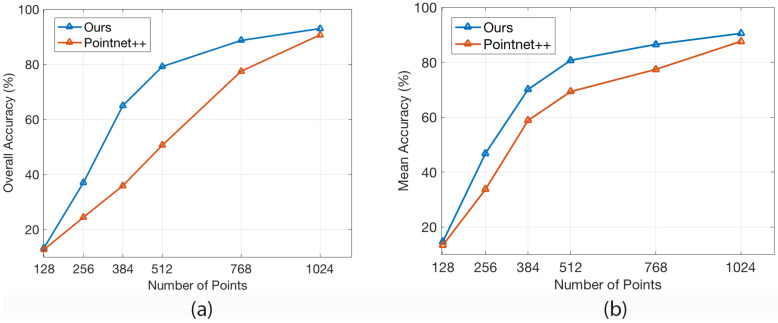
Classification accuracy of our model and PointNet++ with a different number of input points on ModelNet40 test data. (**a**) Overall accuracy across all instances. (**b**) Average of per-class accuracy. The experiments were done without retraining.

We study the robustness of our model to random input dropout compared with PointNet++ without retraining. Figure 6 showed that our approach still can achieve more than 80%+ on both overall and average accuracy of ModelNet40 dataset with only a half of the original number of points. Moreover, the accuracy of our model is significantly better than PointNet++ when the number of input points is between 384 to 768.

#### 4.7.2. Rotation and Translation Perturbations

We also compare the robustness to rotation and translation invariance between our model and PointNet++. The results (Table 1) demonstrate that our model is completely translation invariant and highly robust to the small-range rotation difference with training dataset. There is no point set rotation in data augmentation when training.

## 5. Additional Visualization of Rotated Attention Mask

**Figure 7:**
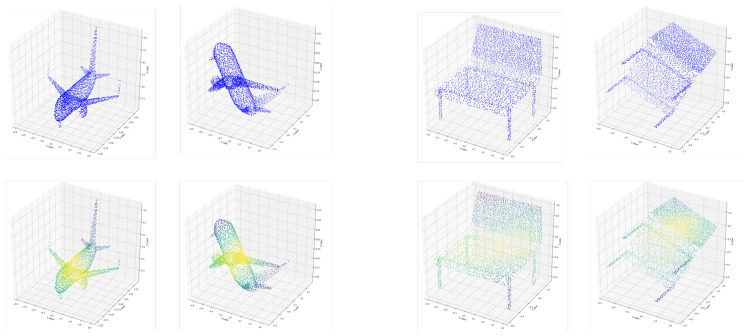
Visualization of rotated structures and attention masks. The first row are original and randomly rotated point sets. The second row are visualization of our generated attention masks respectively. The colors from dark to light correspond to the weights from 1 to 0.

We exploit the influence of rotation of point sets on the generation of our global attention mask. Figure 7 demonstrates that our global attention mask is robust to rotation of point sets. Take the first figure of the plane as an example, although there are some minor difference between two attention masks (such as the the head of the plane), most weight of points in the generated mask remains the same when the input rotates. It still pays more attention to margin areas such as the tail and wings than inner parts of the plane.

## 6. Additional Ablation Study on the ModelNet40 Dataset

**Figure 8:**
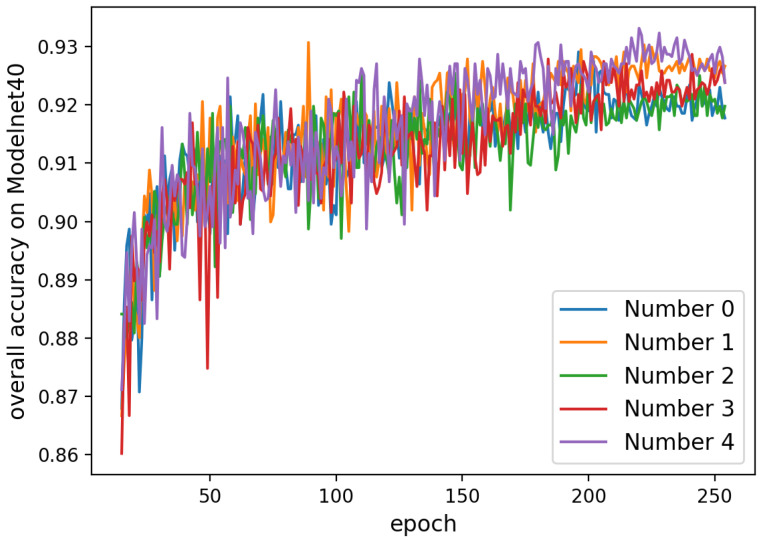
Overall accuracy of different models on ModelNet40 with epochs (epoch >15). The models are defined according to Table 3 of the main text.

**Figure 9:**
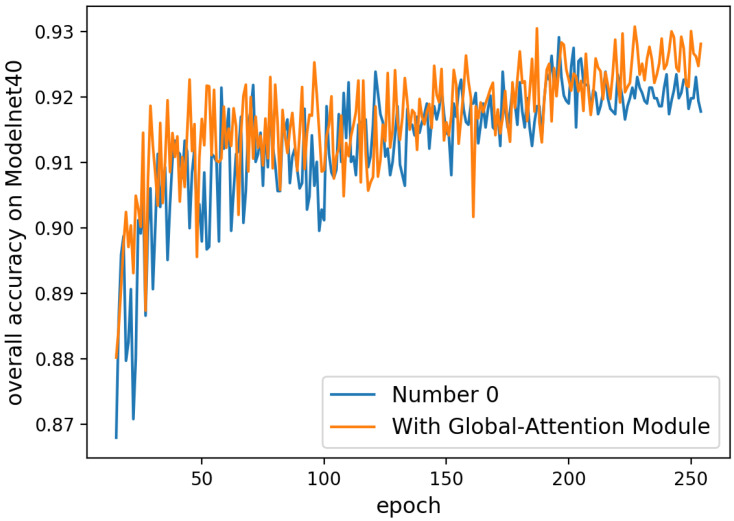
Overall accuracy of different models on ModelNet40 with epochs (epoch >15). Number 0 means removing the Global-Attention Module and all SE-Blocks in our model. With Global-Attention Module denotes that we only remove SE-Blocks in our model.

**Attention Mechanism.** Addition to the examination of our attention mechanism in Section 4.2 and Table 3 of main text, we compare the accuracy of five different models with respect to the number of epochs to demonstrate the general trends. In Figure 8, the model numbers 1 and 4 always achieve high overall accuracy after epoch >220. Except for model number 2, all other models with Spatial-Attention EdgeConvs attain better outcomes than number 0. It demonstrate that the results between models with and without our attention modules have distinct gap after training about 200 epochs and the accuracy does improve by our attention mechanism. In Figure 9, we can see that the accuracy of the model with only Global-Attention Module is always higher than the other which also proves the effectiveness of our design.

**Other Layers.** In Table 6, we demonstrate the model performance in different situations. We consider factors including number of points, batch norm layers [36], dropout layers, activation layers, and aggregation functions. From the results in Table 6, we can see all these factors do improve performance except for increasing the number of input points. The phenomena that the increase in the number of points does not increase the performance has also been observed in other state-of-the-arts work [40].

**Number of points *k* of a group.** In Table 7, we also evaluate the effects of different number *k* of a neighboring point group. Experiments show that small *k* (k≤20) achieves similar accuracy both with 1024 and 2048 input points. By contrast, the performance of large *k* (k≥25) will decrease quickly when having 1024 input points probably because it is hard to find discriminative patterns in large groups. However, more input points (2048 points) will make point sets dense so that groups containing large number of neighboring points may cover regions of same volumes as in sparse point sets with less input points (1024 points), thus maintaining a high accuracy. Besides, despite fed on different input points, k=20 achieves best result 93.6% in both models B2 and B6 in Table 7.

## 7. Conclusions

**Figure 10:**
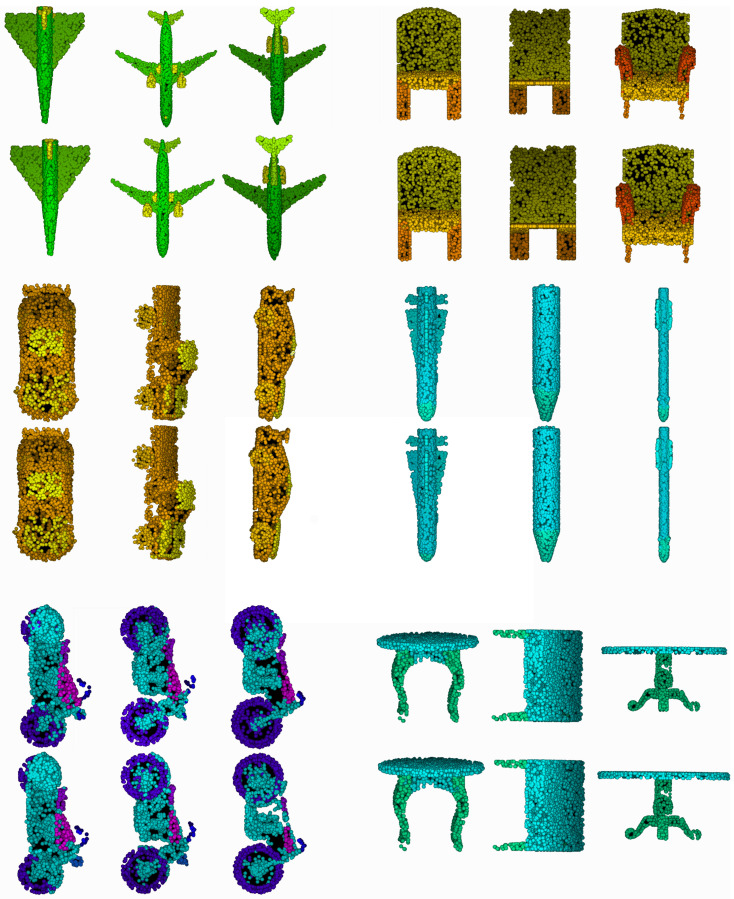
More visualizations of segmentation on ShapeNet dataset. Different segments are represented by different colors. The upper object of a pair is the prediction of AttPNet and the lower one is the ground truth.

In this paper, we propose a novel model named AttPNet that combines a global point-wise attention mechanism and channel weighting to improve performance of point set analysis. AttPNet outperforms the best model in ModelNet40 classification benchmark by 0.7%, which is a significant improvement. In addition, AttPNet is robust to rotational perturbations and missing points. Further experiments also demonstrate that our model performs well on the classification of fine-grained point sets such as the ECT dataset. Furthermore, we provide the visualization of our attention masks on the objects in ModelNet40 and the results of part segmentation in ShapeNet (see Figure 10).

In the future, we will continue optimizing the AttPNet and apply it to other fields such as semantic segmentation. In addition, experiments indicate that there is still considerable potential for improvement in recognizing data with large-angle rotation. Therefore, we will keep working on the robustness of our model. Besides, in many data sets, points are always distributed in an unequal spatial distribution. We will attempt to adapt our model to such attributes in point set and make it focus more on dense areas to attain greater performance.

## Figures and Tables

**Table 1:** Robustness to translation and rotation in terms of classification accuracy. We evaluate the test data with uniform translation in [−0.2,0.2] and the different rotation with $10^{\circ }$, $20^{\circ }$, and $30^{\circ }$. There is no point set rotation in the phase of data augmentation during training.

Method	Translation	R$10^{\circ }$	R$20^{\circ }$	R$30^{\circ }$
Ours	**93.4**	**93.2**	**92.1**	**86.3**
PointNet++	90.6	90.3	88.6	83.8

**Table 2:** Classification results on ModelNet40. (Model-num denotes the model with num layers. +n represents that the input contains normal vectors. OA means the overall accuracy.)

Method	Input	OA (%)
PointNet [3]	1024	89.2
PointNet++ [4]	1024	90.7
PointNet++ [4]	5000 + n	91.9
PointCNN [38]	1024	92.2
DGCNN [16]	1024	92.2
PCNN [39]	1024	92.3
SpiderCNN [20]	1024 + n	92.2
SpiderCNN-4 [20]	1024 + n	92.4
PointConv [19]	1024 + n	92.5
Point2seq [23]	1024	92.6
RS-CNN [40]	1024	92.9
SO-Net-2 [41]	2048	90.9
SO-Net-3 [41]	5000 + n	93.4
Ours (BASELINE)	1024	92.5
Ours (global attention)	1024	92.9
Ours (hard attention)	1024	92.5
Ours (multi-attention)	1024	93.3
Ours (global + channel)	1024	**93.6**

**Table 3:** The overall accuracy (OA) with different number of Spatial-Attention EdgeConv replacing normal EdgeConv. We first remove all SE-Blocks and the Global-Attention Module in Figure 2. Then, we replace different number of common EdgeConv with Spatial-Attention EdgeConv (from left to right in Figure 2).

Number	0	1	2	3	4
OA (%)	92.8	93.1	92.6	92.9	93.3

**Table 4:** Classification results on the ECT dataset.

Method	OA (%)
PointNet [3]	47.78
PointNet++ [4]	94.62
Ours (global + channel)	**96.28**

**Table 5:** Part segmentation results (%) on ShapeNet. Here we list the mean IoU for class and instance. (“*”: add normal vectors with points; “m”: use mesh as input).

Method	Class mIoU	Instance mIoU
PointNet [3]	80.4	83.7
PointNet++ * [4]	81.9	85.1
SpiderCNN [20]	82.4	**85.3**
SPLATNet [13]	82.0	84.6
SyncSpecCNN ${}^{m}$ [44]	82.0	84.7
DGCNN [16]	82.3	85.1
SO-Net * [41]	80.8	84.6
Ours	**82.8**	85.2

**Table 6:** Ablation study of AttPNet on ModelNet40 dataset. “BN” denotes batch norm layers [36]. “DP” represents dropout layers. “Act.” indicates activation layers. “max&avg.” means that we combine the results from two aggregation functions. Model A4 corresponds to AttPNet.

Model	#Points	BN	DP	Act.	Max	Max&Avg.	Acc
A1	1 k			LR		√	90.8
A2	1 k	√		LR		√	91.6
A3	1 k		√	LR		√	93.2
A4	1 k	√	√	LR		√	93.6
A5	1 k	√	√	R		√	92.8
A6	2 k	√	√	LR		√	93.6
A7	1 k	√	√	LR	√		93.3

**Table 7:** Ablation study of AttPNet on ModelNet40 dataset. $N_{knn}$ indicates the number of points in a neighboring group.

Model	#Points	$N_{k\mathrm{nn}}$	Accuracy
B1	1 k	15	93.3
B2	1 k	20	93.6
B3	1 k	25	92.8
B4	1 k	30	92.6
B5	2 k	15	93.5
B6	2 k	20	93.6
B7	2 k	25	93.5
B8	2 k	30	93.5
DGCNN [16]	1 k	5	90.5
DGCNN [16]	1 k	10	91.4
DGCNN [16]	1 k	20	92.9
DGCNN [16]	1 k	40	92.4
